# Exhaustion Experiences in Junior Athletes: The Importance of Motivation and Self-Control Competencies

**DOI:** 10.3389/fpsyg.2016.01867

**Published:** 2016-11-24

**Authors:** Gro Jordalen, Pierre-Nicolas Lemyre, Natalie Durand-Bush

**Affiliations:** ^1^Department of Coaching and Psychology, Norwegian School of Sport SciencesOslo, Norway; ^2^Department of Coaching and Psychology and Norwegian Research Center for Training and Performance, Norwegian School of Sport SciencesOslo, Norway; ^3^School of Human Kinetics, University of Ottawa, OttawaON, Canada

**Keywords:** motivation regulations, self-control, exhaustion, ego-depletion, junior athletes, elite sport development

## Abstract

Quality of motivation, self-control competencies, as well as past performance experience influence sport participation outcomes in developing athletes. Studies have shown that junior athletes high in self-determined motivation are less prone to experience burnout, while self-control competencies help developing athletes to be conscious and deliberate in their self-regulatory efforts toward elite sport performances and avoid negative sport participation outcomes. Combining the self-determination theory framework and psychosocial theories of self-regulation, the aim of this cross-sectional study was to examine how various types of motivation and self-control competencies together are associated with the development of burnout symptoms in junior athletes. High-level Norwegian winter-sport athletes from elite sport academies (*N* = 199; female *n* = 72; 16–20 years of age) consented to participate. Associations between six types of motivational regulation, self-control, and indices of exhaustion were investigated. We hypothesized that athletes’ self-control competencies are important to operate successfully, and influenced by different types of motivation, they are expected to help athletes avoid negative sport participation outcomes such as emotional and physical exhaustion. Structural equation modeling analyses were conducted to analyze these relationships, and results revealed some multifaceted associations. When identifying antecedents of sport participation exhaustion and burnout, there is a need to go beyond the unique framework of motivation theories, and explore what cognitive competencies ensure fulfillment of motivation desires. In the current study, differences in junior athletes’ quality of motivation influenced self-control competencies when predicting exhaustion. Interestingly, young athletes driven by self-determined (intrinsic, integrated, and identified), and controlled (introjected and amotivation) regulations in association with self-control offered the strongest negative and positive associations with exhaustion, respectively. Findings clearly indicate that motivation and self-control competencies are meaningfully interrelated when assessing burnout propensity in young developing athletes.

## Introduction

In Norway, talented junior athletes often attend elite sport academies (e.g., The Norwegian College of Elite Sport), to help facilitate the combination of education and elite sport development, while also preventing overload and maladaptive outcomes such as burnout. Within these academies, athletes belong to an environment focusing on development of expertise and psychological competencies necessary for competing at the highest level. The beginning of the winter-sport season is a key time point where athletes focus on demonstrating competencies. That is, they try to establish themselves as contenders in their sport. This also corresponds to the end of the high school semester where major tests and exams are scheduled. As academies are located in different parts of the country, athletes will often experience challenging situations with limited family support. Hence, the quality of motivation to pursue a sporting career will likely affect developmental outcomes and performance level during these years of athletic development (Ericsson, 2007).

Self-determination theory (SDT; Deci and Ryan, 1985; Ryan and Deci, 2000) states that athletes can be moved and inspired to practice sports by two broader types of motivation, namely self-determined and controlled forms of motivation. Within these broader types, SDT describes motivational regulations along a continuum, ranging from three types of self-determined regulations namely intrinsic, integrated, and identified; two types of controlled regulations, namely introjected and external; and one referring to the absence of regulation namely amotivation. Self-determined forms of motivation refer typically to engagement in an activity driven by fun, genuine interest, personal values, and importance of the activity. More controlled forms of motivation refer to individuals driven by pressure, prods, and external reward (Ryan and Deci, 2000).

Full-time engagement in sport is time-consuming and strenuous, and the importance of engagement due to self-determined reasons is key to healthy youth sport development (Ryan and Deci, 2000). When driven by controlled forms of motivation over a long period of time in combination with increasing signs of amotivation, athletes become more at risk for negative sport participation outcomes such as overtraining and burnout (Lemyre et al., 2007). Though, research agrees that higher levels of self-determined forms of motivation generally increase chances to succeed and reach the elite level of sports (Gillet et al., 2013), some findings prove that title and medal holders can also be driven by higher levels of non-self-determined forms of motivation and amotivation in comparison to less successful athletes (Chantal et al., 1996). Thus, there is an ongoing need to explore the multidimensionality and complexity of motivation and acknowledge the contribution of different motivational regulations within athletes’ motivational profiles believed to influence long-term development. For example, Gillet et al. (2012) found that a profile high in self-determined and controlled forms of motivation resulted in the best performances, but this profile co-occured with higher levels of exhaustion. As such, motivational profiles composed of moderate to high levels of self-determined and moderate levels of controlled forms of motivation might engender both high-level performances and the best psychological adjustment over time (Martinent and Decret, 2015). However, it is important to note that a pure self-determined motivation profile may not exist in highly competitive and achievement driven sports contexts (Gillet et al., 2009). An examination of the functionality of each motivational regulation relative to other psychological competencies is warranted to predict success. For example, self-regulatory competencies are important for having a long-term perspective and stay focused through prolonged efforts for reaching personal goals (Tangney et al., 2004). Nurtured by motivational feelings and beliefs, self-regulatory competencies refer to athletes thoughts’, feelings, and actions developed for the achievement of personal goals (Zimmerman, 1989). Specifically, autonomously motivated self-regulation involves less contradictory thoughts and feelings of conflict and are likely more energizing, whereas feeling pressured to self-regulate may provoke depletion and experiences of exhaustion longitudinally (Muraven, 2008; Tuk et al., 2015).

Self-regulation has been conceptualized as the interplay between controlled and impulsive processes, and has often been confused with self-control (Milyavskaya et al., 2015). Self-control is the effortful subset of self-regulation (Baumeister et al., 2007), defined as the effortful inhibition of impulses or the overcoming of temptations (Milyavskaya et al., 2015). Differences in degree of self-control may lead to both positive (e.g., happiness, more healthy living) and negative (e.g., psychopathological symptoms) outcomes (Tangney et al., 2004). Thus, self-control describes individuals’ capacity to consciously adjust responses toward self- or other-imposed standards (Baumeister et al., 2007). As such, it often represents a conflict between the two closely interacting brain systems controlling emotional and reflexive versus cognitive and reflective thoughts, respectively (Mischel, 2014). When confronted with conflicts between these systems, only one of them can be satisfied at a time, and an exhausting self-control dilemma may emerge. Conversely, a successful resolution of these conflicts enable athletes to effectively resist temptations and conform to requirements in the efforts to accomplish important goals. Athletes’ capacity to engage in effective self-control (e.g., stay true to future plans, work toward goals) varies (Tangney et al., 2004), it requires a great deal of mobilization and energy, and thus may be depleted like a working “muscle” (Baumeister et al., 2007; Fujita, 2011). As such, self-control is likely dependent of limited resources, potentially inducing short-term impairments (ego-depletion) in subsequent self-control efforts. Ego-depletion followed by inadequate recovery has been linked to major negative outcomes such as underachievement and decreased performance, as people within this state may be unable to control themselves effectively (Baumeister and Vohs, 2016). Attaining certain goals by mean of self-control competencies may not necessarily lead to adaptive or functional athletic development (Fujita, 2011). For example, controlling oneself to consistently practice sports without adequate preparation and recovery will likely result in maladaptive development over time. Severely tired athlete will express lower self-control capacity and are more vulnerable to fatigue and ultimately burnout (Baumeister et al., 2007).

Investigating the effects of a self-regulation intervention in student-athletes, Dubuc-Charbonneau and Durand-Bush (2015) found that higher self-regulatory capacity was associated with reduced symptoms of burnout. Burnout in sport has been conceptualized as a multidimensional construct consisting of three dimensions: (a) emotional/physical exhaustion, (b) reduced sense of accomplishment, and (c) sport devaluation (Raedeke and Smith, 2001). These dimensions are characterized by feelings of emotional and physical fatigue caused by training and competition stressors; inefficacy and a tendency to evaluate oneself negatively; and finally negative and detached attitudes toward sports and lack of sport and performance quality concerns, respectively. Associations between athletes’ motivational regulations and burnout propensity have been carefully investigated (e.g., Lemyre et al., 2006; Lonsdale et al., 2009), and negative motivational trends have been associated with increased burnout scores. Being driven by high quality motivation will help developing athletes to flourish and excel, especially when engaged in high-level sports and education simultaneously (Martinent and Decret, 2015). Research has suggested that young student-athletes are at risk for burnout due to the high emotional, physical, and psychosocial demands inherent to their situation (Isoard-Gautheur et al., 2013). Adequate self-control competencies combined with optimal forms of motivation may help athletes avoid burnout symptoms as they get more practice experiences in the ongoing pursuit toward elite level performances.

**FIGURE 1:**
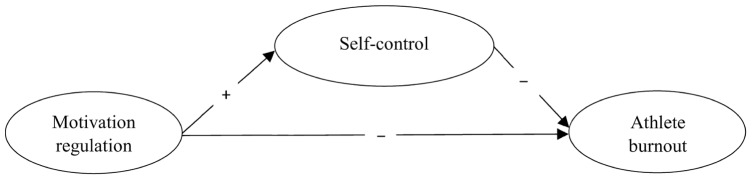
**The hypothesized structural model**.

Relevant practice experiences over time influences athletes’ development and chances to successfully reach the elite level (Ericsson, 2013). Interestingly, some people with unique qualities have been found to reach world-class performance within 6 years (Ericsson, 2006). In addition to practice experiences, the nature of elite competitions and competitive experiences provide athletes with psychological skills necessary for success (Gould et al., 2002). These skills develop throughout an athlete’s career, as athletes with more competitive experiences have a greater chance of learning key psychological skills necessary for success (e.g., appropriate focus, self-control). In Norway, children are allowed to compete at the age of six, while they cannot be ranked before they are 11 years old in most sports. Hence, from the age of eleven they will acquire the more genuine experiences of skiing competitions in Norway. However, junior athletes developing exceptional skills will likely struggle without motivation as well as self-control to train and compete at the highest level. These concepts have been extensively studied in the sport context, but no study has addressed the complexity of athletes’ motivation in association with the quality of self-control competencies to predict sport participation outcomes. As such, the current study examines associations between the type of motivation, self-control, and symptoms of burnout in junior Norwegian winter sport athletes (**Figure 1**). We hypothesized that the associations between athletes’ self-control competencies and symptoms of burnout are dependent on different motivational regulations. That is, more self-determined types of motivation will energize self-control competencies, and when combined they will yield a negative association to burnout. On the other hand, more controlled forms of motivation will induce ego-depletion and offer a positive association to burnout.

## Materials and Methods

### Participants

A total of 199 winter sport athletes (123 male, 72 female, and 4 did not report gender; 16–20 years of age, *M* = 17, *SD* = 0.97) attending elite sport colleges in Norway consented to participate. Participants provided written informed consent in accordance with the Declaration of Helsinki. Athletes competed in cross-country skiing (*n* = 51), biathlon (*n* = 68), ski jumping (*n* = 53), alpine skiing (*n* = 22), and some athletes (*n* = 5) did not indicate their main sport. Competitive experiences ranged from 1 to more than 15 years (*M* = 6.83 years), and athletes competed at international (*n* = 23), national (*n* = 153), or regional levels (*n* = 17).

### Measures

#### Motivation

The Sport Motivation Scale II (SMS-II; Pelletier et al., 2013) measured athletes’ motivational regulations, and response options ranged from 1 (*does not correspond at all*) to 7 (c*orresponds completely*). The questionnaire was translated using the translation-back translation method (Brislin, 1970). That is, the first author familiar with both languages translated the original questionnaire, and two bilingual colleagues performed back translation. Then, the back translated questionnaires were compared, checked for equivalence to the original questionnaire, and necessary adjustments were made. Further, latent variable modeling was used to evaluate scale reliability [coefficient *rho* (ρ)], and validity coefficients in the structural equation modeling (SEM) analyses added reliability information (see **Table 1**; Brown, 2006; Raykov, 2009). Assumptions for alpha reliability are likely violated in empirical research (Raykov, 2009; Yang and Green, 2011), thus alternative reliability scores were evaluated in the current study as more accurate reflections of reliability. The assessed regulations were intrinsic (three items, ρ = 0.73; 95% CI = [0.64–0.81]; SE = 0.04; e.g., “because it is very interesting to learn how I can improve”), integrated (three items, ρ = 0.68; 95% CI = [0.57–0.78]; SE = 0.06; e.g., “because participating in sport is an integral part of my life”), identified (three items, ρ = 0.72; 95% CI = [0.63–0.80]; SE = 0.04; e.g., “because I have chosen this sport as a way to develop myself”), introjected (three items, ρ = 0.61; 95% CI = [0.51–0.71]; SE = 0.05; e.g., “because I feel better about myself when I do”), external (three items, ρ = 0.65; 95% CI = [0.52–0.75]; SE = 0.05; e.g., “because people around me reward me when I do”), and amotivated (three items, ρ = 0.82; 95% CI = [0.76–0.87]; SE = 0.03; e.g., “it is not clear to me anymore, I don’t really think my place is in sport”).

**Table 1:** Descriptive statistics and correlations for the study variables^2^.

Variable	*M*	*SD*	1	2	3	4	5	6	7	8
(1) INT	5.98	0.92	0.91							
(2) INE	5.63	1.03	0.78ˆ***	0.91						
(3) IDE	5.45	1.05	0.72ˆ***	0.69ˆ***	0.89					
(4) INR	4.40	1.24	0.40ˆ**	0.64ˆ***	0.44ˆ**	0.87				
(5) EXT	2.32	1.04	-0.18	0.06	0.03	0.54ˆ***	0.91			
(6) AMO	2.39	1.51	-0.55ˆ***	-0.48ˆ***	-0.10	0.13	0.55ˆ***	0.93		
(7) SC	3.60	0.64	0.42ˆ***	0.23ˆ*	0.21	-0.29ˆ*	-0.41ˆ***	-0.51ˆ***	0.92	
(8) EX	1.98	0.77	-0.31ˆ**	-0.18	0.04	0.14	0.40ˆ***	0.41ˆ***	-0.51ˆ***	0.94

*INT, Intrinsic regulation; INE, Integrated regulation; IDE, Identified regulation; INR, Introjected regulation; EXT, External regulation; AMO, Amotivation; SC, Self-control; EX, Exhaustion. Motivation regulation, self-control, and exhaustion mean validity coefficients at the diagonal (recommended score >0.80; Brown, 2006). ^∗^*p* < 0.05; ^∗∗^*p* < 0.01; ^∗∗∗^*p* < 0.001.*

*^2^Correlations were performed in M*plus* version 7.31. Means of latent variables are zero in cross-sectional studies; hence, descriptive statistics was performed in IBM SPSS Statistics 21.*

#### Self-Control (SC)

A Norwegian version of the Brief Self-Control Scale (BSCS; Tangney et al., 2004) assessed the athletes’ dispositional SC abilities (12 items, ρ = 0.83; 95% CI = [0.79–0.87]; SE = 0.02; e.g., “I am good at resisting temptations”). Response options ranged from 1 (*not at all*) to 5 (*very much*). Items 2, 3, 4, 5, 7, 9, 10, 12, and 13 were reverse scored (Tangney et al., 2004). Item 6 was deleted due to low factor loading (< 0.50; Kline, 2011).

#### Athlete Burnout

A Norwegian version (Lemyre et al., 2006) of the Athlete Burnout Questionnaire (ABQ; Raedeke and Smith, 2001) assessed athlete burnout. The ABQ is a sport-specific multidimensional measure composed of three subscales measuring emotional and physical exhaustion (ABQE; five items, ρ = 0.85; 95% CI = [0.81–0.90]; SE = 0.02; e.g., “I feel ‘wiped out’ from [sport]”), reduced sense of accomplishment (ABQR; five items, ρ = 0.72; 95% CI = [0.64–0.79]; SE = 0.04; e.g., “I am not achieving much in [sport]”), and sport devaluation (ABQD; five items, ρ = 0.77; 95% CI = [0.72–0.82]; SE = 0.03; e.g., “I’m not into [sport] like I used to be”). Response options ranged from 1 (*almost never*) to 5 (*almost always*). Items 1 and 14 were reverse scored.

### Procedures

Subsequent to approval by the Norwegian Centre for Research Data (NSD), national ethical standard procedures were followed for the protection of research participants. In the recruitment phase, sports directors at elite sport colleges in Norway were contacted, and following approval from these directors athletes were invited to participate. The information letter, declaration of consent, and questionnaires were delivered and returned by e-mail and mail, and the survey was arranged at the beginning of the competitive season. Hence, data collection was completed when athletes experienced a challenging period (e.g., they wanted to prove sport performance progress) and the concepts under study are especially meaningful.

### Statistical Analyses

Confirmatory Factor Analyses (CFA) in M*plus* Version 7.31 (Muthén et al., 1998-2016) were performed, and variables’ model fit evaluated. That is, six motivational regulations tested individually, self-control composed of six parcels, and athlete burnout composed of three indicators’ (i.e., emotional and physical exhaustion, reduced sense of accomplishment, and sport devaluation) represented the latent variables motivation, self-control, and burnout. Parceling self-control items to manifest indicators by means of the balancing approach is advantageous due to psychometric characteristics and model estimation procedures (Little, 2013). Model identification was achieved by fixing one item-factor loading per latent variable to 1.0, and model fit was determined by various Goodness-of-fit (GOF) indices (Kline, 2011; Byrne, 2012): the χ^2^, RMSEA combined with its 90% CI, CFI, and the SRMR. Traditional cutoff criteria (CFI: 0.90–0.99, RMSEA: 0.08–0.05, and SRMR ≤ 0.08) indicated acceptable fit (Brown, 2006; Little, 2013, p. 109). However, researchers must use caution using these GOF indices, aiming for modification indices (MI) < 10, and ideally, factor loadings > 0.50 (Brown, 2006; Kline, 2011; Byrne, 2012). Missing data (< 5.0%) were handled using the full information maximum likelihood (FIML) estimation, and analyses were performed using the robust MLR-estimator (Enders, 2010).

First, using SEM analyses, we examined associations between motivational regulations, self-control and athlete burnout among athletes in the total sample, testing the indirect effect of self-control in the motivation to burnout association. Though, it has been debated whether a mediation model based on cross-sectional data without the possibility of looking at longitudinal causal effects is valuable (Jose, 2016). However, the ordering of variables is based on previous research (e.g., Lemyre et al., 2006; Mischel, 2014). That is, self-determined types of motivation are likely to increase athletes’ self-control capacity and hence result in decreased symptoms of burnout, and conversely, controlled types of motivation are likely to decrease athletes’ self-control capacity and hence result in negative development and increased symptoms of burnout. Additionally, the resampling procedure called bootstrapping used in the current study has recently showed valid results (Hayes, 2009), and is preferred above the Sobel’s test because it is more informative (Hayes and Scharkow, 2013; Jose, 2016).

## Results

In the SEM analyses, three indicators were specified defining the motivation regulations and burnout latent constructs, thereby meeting indicator requirements for one-factor models (Brown, 2006). Evaluating fit indices for the six motivational regulations model resulted in acceptable fit, χ^2^(120) = 209.91, *p* < 0.05, RMSEA = 0.06, 90% CI [0.05, 0.08], SRMR = 0.08, and CFI = 0.90. However, due to this model’s complexity and the sample size of the current study, the motivation regulations were evaluated individually in six different models (Kline, 2011). In the resulting six one-factor models for motivation regulations the GOF evaluation does not apply because these models are just-identified (Brown, 2006). However, models were evaluated based on interpretability and strength of parameter estimates (factor loadings), ranging from 0.39 to 0.90, explaining 15–80% of the variance. The latent construct representing self-control (parcels)^1^ showed good fit, χ^2^(9) = 15.87, *p* > 0.05, RMSEA = 0.06, 90% CI [0.00, 0.11], SRMR = 0.03, and CFI = 0.97. Evaluating model fit for the burnout subscales individually, the exhaustion and reduced sense of accomplishment burnout subscales showed acceptable fit, χ^2^(5) = 9.32, *p* > 0.05, RMSEA = 0.07, 90% CI [0.00, 0.13], SRMR = 0.03, and CFI = 0.98; and, χ^2^(5) = 10.98, *p* > 0.05, RMSEA = 0.08, 90% CI [0.00, 0.14], SRMR = 0.05, and CFI = 0.94, respectively. However, the devaluation subscale showed non-acceptable fit, χ^2^(5) = 29.05, *p* < 0.05, RMSEA = 0.16, 90% CI [0.11, 0.22], SRMR = 0.06, and CFI = 0.86, and hence was excluded from further analyses. Thus, based on conceptual arguments that self-control is more related to depletion patterns (Baumeister and Vohs, 2016), the reduced sense of accomplishment burnout subscale was excluded from the analyses, and motivation regulation → self-control → emotional and physical exhaustion associations were evaluated.

**Table 1** presents correlations between the study variables and descriptive statistics. Self-control and intrinsic, integrated, and identified regulations were positively associated; and negatively associated with exhaustion. Introjected and external regulations, and amotivation were negatively associated with self-control; and positively associated with exhaustion. Additionally, self-control and exhaustion were negatively associated. Further, mean scores were high for intrinsic, integrated, and identified regulations; moderate for introjected regulation and self-control; and low for external regulation, amotivation, and exhaustion.

Model fit results for the structural equation models are presented in **Table 2**. This table additionally presents model fit results for the 95% bias-corrected CI derived from 10.000 resamples (Hayes and Scharkow, 2013), examining direct and indirect effects between latent construct. Total effects are reported as the unmediated associations between motivation and exhaustion, direct effects as the mediated associations between motivation and exhaustion, and indirect effects as the estimated effect of self-control in the motivation → exhaustion association (Jose, 2016). Further, effects evaluated in the current study are often evident only in the estimate’s confidence interval and not in the *p*-value. Thus, note that *p*-values are sample size sensitive and researchers need to evaluate additional criteria when judging the importance of findings (Ivarsson et al., 2013). In the first SEM analysis testing intrinsic regulation → self-control → exhaustion associations, standardized showed significant total and indirect effects (estimate = -0.28, SE = 0.11, 95% CI [-0.49, -0.05], *p* = 0.014; and estimate = -0.19, SE = 0.06, 95% CI [-0.35, -0.10], *p* = 0.002; respectively), and a non-significant direct effect (estimate = -0.09, SE = 0.13, 95% CI [-0.34, 0.16], *p* = 0.508). In the second structural model testing integrated regulation → self-control → exhaustion associations, standardized results showed non-significant total and direct effects (estimate = -0.17, SE = 0.10, 95% CI [-0.36, 0.04], *p* = 0.095; and estimate = -0.06, SE = 0.10, 95% CI [-0.24, 0.13], *p* = 0.546, respectively), though a significant indirect effect (estimate = -0.11, SE = 0.06, 95% CI [-0.24, -0.02], *p* = 0.040). In the third structural model testing identified regulation → self-control → exhaustion associations, standardized results showed non-significant total and direct effects (estimate = 0.05, SE = 0.10, 95% CI [-0.14, 0.24], *p* = 0.586; and estimate = 0.17, SE = 0.09, 95% CI [-0.02, 0.35], *p* = 0.075, respectively), and a significant indirect effect (estimate = -0.11, SE = 0.07, 95% CI [-0.26, -0.01], *p* = 0.083). In the fourth structural model testing introjected regulation → self-control → exhaustion associations, standardized results showed non-significant total and direct effects (estimate = 0.15, SE = 0.12, 95% CI [-0.06, 0.39], *p* = 0.206; and estimate = -0.01, SE = 0.11, 95% CI [-0.20, 0.22], *p* = 0.907, respectively), though a significant indirect effect (estimate = 0.16, SE = 0.06, 95% CI [0.06, 0.32], *p* = 0.013). In the fifth structural model testing external regulation → self-control → exhaustion associations, standardized results showed significant total, direct, and indirect effects (estimate = 0.41, SE = 0.09, 95% CI [0.24, 0.58], *p* = 0.000; estimate = 0.25, SE = 0.11, 95% CI [0.03, 0.46], *p* = 0.023; and estimate = 0.16, SE = 0.05, 95% CI [0.08, 0.29], *p* = 0.002, respectively). Finally, in the sixth structural model testing amotivation → self-control → exhaustion associations, standardized results showed significant total and indirect effects (estimate = 0.39, SE = 0.09, 95% CI [0.21, 0.56], *p* = 0.000; and estimate = 0.21, SE = 0.06, 95% CI [0.10, 0.36], *p* = 0.001, respectively), though non-significant direct effects (estimate = 0.19, SE = 0.12, 95% CI [-0.06, 0.43], *p* = 0.134).

**Table 2:** Structural and bootstrapped model results.

Model	χ^2^(df)	*p*-value	RMSEA	90% CI	SRMR	CFI
1 Structural	110.92(74)	0.0035	0.05	[0.03, 0.07]	0.06	0.95
1 Bootstrapped	125.44(74)	0.0002	0.06	[0.04, 0.08]	0.06	0.95
2 Structural	113.52(74)	0.0021	0.05	[0.03, 0.07]	0.05	0.95
2 Bootstrapped	119.58(74)	0.0006	0.06	[0.04, 0.07]	0.05	0.95
3 Structural	92.94(74)	0.0675	0.04	[0.00, 0.06]	0.05	0.98
3 Bootstrapped	98.17(74)	0.0316	0.04	[0.01, 0.06]	0.05	0.97
4 Structural	115.67(74)	0.0014	0.05	[0.03, 0.07]	0.07	0.94
4 Bootstrapped	126.62(74)	0.0001	0.06	[0.04, 0.08]	0.07	0.94
5 Structural	95.04(74)	0.0503	0.04	[0.00, 0.06]	0.05	0.97
5 Bootstrapped	103.59(74)	0.0132	0.05	[0.02, 0.06]	0.05	0.97
6 Structural	96.67(74)	0.0397	0.04	[0.01, 0.06]	0.05	0.97
6 Bootstrapped	104.84(74)	0.0106	0.05	[0.02, 0.07]	0.05	0.97

*Model 1, Intrinsic regulation → Self-control → Exhaustion; Model 2, Integrated regulation → Self-control → Exhaustion; Model 3, Identified regulation → Self-control → Exhaustion; Model 4, Introjected regulation → Self-control → Exhaustion; Model 5, External regulation → Self-control → Exhaustion; Model 6, Amotivation → Self-control → Exhaustion. We used the MLR-estimator in Structural models, and 95% bias-corrected bootstrap CI derived from 10.000 resamples in bootstrapped models.*

## Discussion

In the current study, motivational regulations and self-control competencies were combined to look at the association with emotional and physical exhaustion. Direct associations among motivation and burnout subscales have been tested previously (e.g., Lemyre et al., 2006), and the depletion effect of self-control has recently been extensively debated (e.g., Carter et al., 2015; Baumeister and Vohs, 2016; Hagger and Chatzisarantis, 2016). Interestingly, there has been suggestions that ego-depletion effects following acts of self-control are related to individuals type of motivation (e.g., Inzlicht and Schmeichel, 2012). However, these associations have not been studied in-depth. An examination of the indirect effect of self-control on the motivation to exhaustion association offers the potential to extend our current state of knowledge on processes affecting burnout propensity in young developing athletes. As such, analyses investigated whether the functionality of self-control was dependent on types of motivation regulation (e.g., intrinsic vs. external), and whether associations between motivation and self-control competencies generate negative outcomes such as exhaustion experiences in junior athletes.

In general, athletes reported high levels of self-control and self-determined motivation (i.e., intrinsic, integrated, and identified regulations), moderate levels of introjected regulation, and low levels of external regulation, amotivation, and exhaustion. Consistent with former research (Li et al., 2013), higher levels of self-determined regulations were negatively associated with exhaustion, whereas more controlled types of motivational regulation (amotivation, introjected, and external regulations) were positively associated with exhaustion. Noteworthy, the identified motivation regulation was positively associated with exhaustion. This is not in line with previous research (e.g., Lonsdale et al., 2009), and reflects that highly competitive elite sport performers may show a different motivational profile compared to performers in other contexts (Gillet et al., 2009).

Direct motivation to exhaustion associations remained, respectively, negative and positive when testing self-determined (intrinsic and integrated) and controlled (external and amotivation) forms of motivation regulation. Further, the identified and introjected regulations were, respectively, positively and negatively associated with exhaustion in the direct association. These results are conceptually (Ryan and Deci, 2000) and scientifically (e.g., Lonsdale et al., 2009) different from former publications, reasonably due to the powerful self-control indirect effect. Combined with self-determined forms of motivation (i.e., intrinsic, integrated, and identified regulations) self-control was negatively associated with exhaustion, and combined with controlled forms of motivation, self-control was positively associated with exhaustion. Interestingly, the external regulation persistently showed a significant negative direct association to exhaustion, whereas the intrinsic, integrated, identified, introjected, and amotivation regulations were more complex as they showed the most powerful and significant associations with exhaustion through self-control. The most and the least self-determined types of motivation are strong predictors and reflect humans’ natural propensity to learn and assimilate, on the one side, and to be externally controlled without true self-regulation, one the other (Ryan and Deci, 2000). The most self-determined forms of motivation are characterized by fun, intrinsic interest, and enjoyment, while the least self-determined forms of motivation are associated with a lack of control and intention, and engagement due to external reward. Even though these types of motivation are strong individual predictors, it seems that in the current study they are more influenced by athletes’ cognitive competencies and not solely responsible for an athlete’s initiatives. Conversely, integrated, identified, and introjected regulations are characterized by personal importance, conscious valuing, and engagement due to internal reward (Ryan and Deci, 2000). Intuitively and in accordance with current study findings, they seem to be more influenced by self-control competencies, reflecting the necessity of self-regulatory efforts to successfully operate. Thus, as self-control competencies combined with self-determined motivation are negatively related to exhaustion, this might suggest that self-control does not automatically cause depletion patterns (Carter et al., 2015; Baumeister and Vohs, 2016; Hagger and Chatzisarantis, 2016).

Extensive interest in studying self-control among social psychologists began in the early 2000s (Inzlicht and Schmeichel, 2012), when Baumeister et al. (1998) introduced the strength model of self-control. In their model, self-control is relying on limited physiological and cognitive resources, thus acts of self-control lead to depletion (i.e., ego-depletion). However, recent research has questioned the ego-depletion effect, and findings suggest that this effect seems clearer when self-control is executed sequentially rather than executed on several tasks simultaneously (Tuk et al., 2015). Furthermore, studying psychological phenomena in laboratory experimental research settings might have been limiting and may be the cause for some vague findings (Carter et al., 2015; Baumeister and Vohs, 2016). Thus, research needs to provide a more nuanced picture on whether self-control and executive functions deplete individuals physiological and cognitive resources, and the suggestion that patterns of depletion are influenced by individuals motivational regulations appear promising (Inzlicht and Schmeichel, 2012). In the current study, self-control combined with more controlled forms of motivation (introjected, external, and amotivation) were linked to symptoms of exhaustion and eventually depletion patterns. These findings suggest that high self-control capacity combined with self-determined forms of motivation helps junior athletes avoid maladaptive experiences of overload and burnout. Athletes may experience more successful recovery and lower levels of stress due to self-control and other cognitive competencies, which enables a better adjustment and possibly lower vulnerability of burnout experiences (Martinent and Decret, 2015). Thus, athletes high in self-determined forms of motivation and self-control may resist temptations and stay with practice activities and long-term goals in order to achieve delayed gratifications in the form of good health, development, and eventually great performances (Tangney et al., 2004). However, understanding the complexity of motivation needs further elaborations on exploring the various forms of motivation regulation in detail. For example, why does the direct link between introjected and identified regulations with exhaustion end up slightly negative and positive, and why did indirect effects of self-control result in positive and negative associations toward the maladaptive outcome of exhaustion? Results seem to emphasize that in order to understand how self-control is facilitated by motivational desires require a detailed and inclusionary examination of these related concepts (Baumeister, 2016). In summary, results confirm our hypothesis that self-control competencies seem to depend on the type of motivation initiating behaviors, and when investigating patterns of human motivation researchers need to consider humans’ executive functioning (Vohs et al., 2014).

Exercised successfully, individuals’ self-control capacity seems to be dependent on the type of motivation initiating behaviors. This underlines the complexity of motivation in highly competitive samples. For example, how controlled types of motivation inspire self-control competencies and increase the vulnerability for exhaustion experiences (Gillet et al., 2009, 2012). This complexity may originate in the fact that athletes performance motivation contain self-determined and controlled forms of motivation simultaneously (Martinent and Decret, 2015). On the one side, athletes strive to reach the elite level of performance because it is intrinsically interesting and fun, and on the other side, they want to prove that they are skillful and strive for acceptance and recognition from others (Ryan and Deci, 2000). As such, successful athletes seem to use the interaction between various forms of motivation and cognitive competencies in their ongoing drive for outstanding results. Athletes’ type of motivation originates in basic drives to develop successfully, and self-control and other cognitive competencies further facilitate athletes’ motivation (Hofmann et al., 2012; Baumeister, 2016). Though, experiences of burnout may develop over time (Madigan et al., in press), and the contribution of various motivational regulations combined with self-control competencies reflects that athletes are walking a fine line when it comes to developmental outcomes. Thus, high levels of motivation might increase the risk for exhaustion and burnout experiences over time (Lemyre et al., 2008). Athletes driven by moderate levels of self-determined and controlled motivation simultaneously might be especially vulnerable for psychological maladjustment, as they might experience more sport-specific stress, symptoms of burnout, and additionally poor overall recovery (Martinent and Decret, 2015).

In summary, results from the current study reaffirm the importance of quality of motivation when examining exhaustion experiences and athlete burnout (Cresswell and Eklund, 2005), and show important contributions of self-control in the relationship between these facets of performance. In a more nuanced perspective, findings suggest that self-determined types of motivation energize athletes’ cognitive competencies and negatively predict exhaustion, though the order and direction of these associations need to be further evaluated through longitudinal research. The relationship between motivation and burnout may be reciprocal, and are likely influenced by athletes’ personal disposition (Madigan et al., in press). As such, motivation and self-control competencies should be considered in junior athlete development in order to prevent maladaptive sport participation outcomes.

### Limitations

While this study makes a unique contribution to the literature, findings should be interpreted with caution given the study’s cross-sectional nature, its limited sample size, and self-reported data (Breckler, 1990; Podsakoff et al., 2003; Jose, 2016). Based on the cross-sectional data, the causality of effects investigated could not be stated (Jose, 2016). That is, temporality between variables is the only true way to assess causality, as the independent variable occurs before the mediator, and the mediator occurs before the dependent variable. However, the preferred ordering presented in this article is based on prior research investigating associations between motivation and athlete burnout (e.g., Lemyre et al., 2006), and the evidence that self-control capacity may result in successful or unsuccessful development (Tangney et al., 2004; Mischel, 2014). Further, translation of the SMS-II may have caused linguistic or cultural misinterpretations (Benítez et al., 2016), and the wording of items is not necessarily suitable in a highly competitive Norwegian winter sport context (e.g., item 1, “because it gives me pleasure to learn more about my sport”). In addition, the self-control and ABQs included reverse scored items and may cause method bias (Podsakoff et al., 2003); and some subscales’ validities were questionable (3 out of 10 reliability coefficients were marginal, ranging from 0.61 to 0.68; see “Materials and Methods” section). The self-control and the sport devaluation subscale of the ABQ showed some limitations when it comes to factor structure, as they initially did not reflect acceptable model fit. A careful investigation of these questionnaires in junior athletes is wanted. The BSCS’s unidimensionality and validity has previously been investigated (e.g., Maloney et al., 2012; Toering and Jordet, 2015), however, results from the current study suggest that further revisions might be needed. In the current study, high factor determinacies (ranging from 0.87 to 94; recommended value >0.80) reflected that the factors (i.e., latent constructs) were well measured and acceptable (Muthén et al., 1998-2016; Brown, 2006).

### Future Directions

The model investigated in the current study is novel, but its cross-sectional nature leads to some limitations. Future research needs to investigate associations longitudinally, involving temporality in the mediation analysis. Only then, the causal processes among variables will be truly investigated, and the placement of variables will guide the temporal relations (Jose, 2016). Additionally, examining factor structures of established questionnaires’ reliability and validity may reveal fragile instruments (Clark and Watson, 1995), and based on results from the current study the athlete burnout and self-control questionnaires need to be further evaluated and validated in a Norwegian youth sport setting. Investigating the combination of motivation regulations and cognitive competencies, and going beyond laboratory settings to investigate the self-control depletion phenomenon in elite sport natural settings, is important to understand the complexity of youth sport development (Baumeister, 2016; Baumeister and Vohs, 2016). Further, findings from the current study suggest that athletes’ motivation will benefit from well-developed self-control competencies. As such, longitudinal studies in the domain of individual and team sports are required to extend these findings, and look into athletes’ self-control competencies to better understand the causes of self-control depletion.

## Conclusion

This study showed that various types of motivation combined with self-control competencies are central concepts when identifying antecedents of exhaustion and ego-depletion experiences in junior athletes. The outcome of exercising self-control seems to depend on the type of motivation initiating behaviors, and research needs to consider both a nuanced picture of athletes’ motivation and their cognitive competencies to capture the complexity of youth sport development. Interestingly, the association between motivation, self-control competencies, and exhaustion was more significant compared to the association between motivation and exhaustion directly. As such, well-developed self-control competencies driven by self-determined motivation seem to offer great benefits for junior athletes.

## Author Contributions

All authors listed, have made substantial, direct and intellectual contribution to the work, and approved it for publication.

## Conflict of Interest Statement

The authors declare that the research was conducted in the absence of any commercial or financial relationships that could be construed as a potential conflict of interest.

## Footnotes

1 The original version of the self-control scale showed non-acceptable fit, χ^2^(54) = 118.60, *p* < 0.05, RMSEA = 0.08, 90% CI [0.06, 0.10], SRMR = 0.06, and CFI = 0.86.
